# Guillain-Barré syndrome after tetanus toxoid, reduced diphtheria toxoid and acellular pertussis vaccine: a case report

**DOI:** 10.1186/1752-1947-5-502

**Published:** 2011-10-05

**Authors:** Hussam Ammar

**Affiliations:** 1 Internal Medicine Department, University at Texas, Health Science Center at Houston, Fannin, Houston, TX 77030, USA

## Abstract

**Introduction:**

The association of Guillain-Barré syndrome with vaccination has been described in the literature; it is infrequent and controversial. An association with swine influenza, influenza, hepatitis and tetanus vaccination has been documented in few case reports.

**Case presentation:**

A 40-year-old Caucasian man sustained a small right temporal epidural hematoma and nondisplaced right skull fractures after a fall from a roof. He was managed conservatively; a tetanus toxoid, reduced diphtheria toxoid and acellular pertussis vaccine was administered and a week later he was discharged home. A few days after his discharge, he experienced weakness and numbness in his legs, which progressed to involve his arms. Three weeks after his initial fall, he was readmitted with quadriparesis. A lumbar puncture revealed a cerebrospinal fluid protein of 790 mg/dL and one white blood cell. We diagnosed Guillain-Barré syndrome. Our patient was treated with intravenous immunoglobulin. Three months later his muscle strength had improved, but he continued to have tingling in his hands and feet and used a walker intermittently.

**Conclusion:**

To the best of our knowledge, this is the first case of Guillain-Barré syndrome to be reported in the English literature after administration of tetanus toxoid, reduced diphtheria toxoid and acellular pertussis vaccine.

## Introduction

The association of Guillain-Barré syndrome (GBS) with vaccination has been described but is infrequent and, in many settings, is controversial. A small but statistically significant elevated risk of GBS was noted among swine flu vaccinees relative to non vaccinees in 1976 [1].

The influenza vaccine in the 1992 to 1994 vaccine campaign was associated with a very small increased risk, of one GBS case per million vaccinees. Hepatitis vaccinations were also suggested to be associated with GBS [1]. The association of tetanus vaccination and GBS is documented in only a few case reports (1-5).

## Case presentation

A 40-year-old Caucasian man fell from a second story roof. He sustained a small right temporal epidural hematoma, a nondisplaced right temporal scalp fracture and a zygomatic arch fracture. His neurological examination was normal.

A tetanus toxoid, reduced diphtheria toxoid and acellular pertussis vaccine (Tdap; Boostrix) was administered. He was managed conservatively and discharged home one week later.

A few days after his discharge, our patient experienced weakness and numbness in his legs, which progressed to involve his arms. Three weeks after his initial fall, he was readmitted to our hospital with severe weakness. His muscle strength was 2/5 in his lower extremities and 3/5 in his upper extremities. His deep tendon reflexes were absent at his ankles and knees and were diminished in his upper extremities. There was bilateral peripheral facial paresis, bulbar weakness and impaired gag reflex. He had diminished touch and pin prick sensation in a stocking glove distribution.

His vital capacity was reduced to 1.4 liters. Magnetic resonance imaging of his brain and spinal cord were unremarkable. A lumbar puncture revealed a cerebrospinal fluid protein of 790 mg/dL and one white blood cell.

We diagnosed GBS. Nerve conduction studies revealed prolongation of the upper and lower motor action potential latencies, reduced motor conduction velocities and reduced amplitude. Median and ulnar nerves sensory action potentials were absent. Our patient was treated with intravenous immunoglobulin and his muscle strength improved. Three months later he continued to have tingling in his hands and feet and use a walker intermittently.

## Discussion

The association of tetanus toxoid containing vaccines and GBS is documented in a small number of case reports, outlined in Table 1[1-5].

**Table 1:** Cases describing the association of tetanus vaccination and GBS.

Case	Tetanus containing vaccine	Onset of symptoms after vaccination	Clinical presentation	Treatment	Outcome
[3]	Tetanus toxoid (1962,1971,1976)	First episode: three weeks	First episode: Progressive limb weakness. CSF was normal except a protein level of 300 mg/dL.	Symptomatic treatment and physical therapy.	First episode: Complete recovery after two months.
		Second episode: two weeks	Second episode: Tingling and numbness, mild weakness, loss of position sense, absent DTR. CSF was normal except a protein level of 250 mg/dL.		Second episode: Recovery after eight months with slight hand tremors.
		Third episode: 10 days	Third episode: Tingling and numbness, unable to walk, loss of position sense and diminished pin prick sensation below knees.		Third episode: Patient had only mild hand weakness by day 12.

[4]	Tetanus toxoid.	Nine days	Tingling and numbness, profound weakness of the extremities, bilateral peripheral facial paresis, bulbar weakness, bilateral weakness of ocular abduction. DTR were absent. Touch and pin prick sensation were diminished distally in lower extremities. Vital capacity was 1.6 L. CSF protein level was 550 mg/dL, no pleocytosis.	Symptomatic treatment and physical therapy	Discharge after 57 days with mild left leg weakness.

[5]	Tetanus toxoid.	Four days	Tingling of finger tips and toes, moderate weakness of four extremities. DTR were absent at ankles, knees and triceps. There was bilateral facial weakness and dysphagia. Position and vibration sense was diminished distally in four extremities. CSF protein level was 70 mg/dL.	Intravenous immunoglobulin and physical therapy	Significant improvement at six weeks. Residual facial weakness and painful twitching in both arms.

CSF: cerebrospinal fluid; DTR: deep tendon reflexes

The Advisory Committee on Immunization Practice considers development of GBS less than six weeks after receiving a tetanus toxoid-containing vaccine a precaution for subsequent tetanus toxoid-containing vaccinations.

The Institute of Medicine favored the acceptance of causal relationship between tetanus toxoid-containing vaccine and GBS based on a single documented case report [2]. A subsequent analysis estimated the number of cases of GBS observed after a tetanus toxoid-containing vaccine to be less than the number caused by chance alone [1,2].

Our patient didn't have diarrhea, fever, cough or chills in the weeks preceding his illness. The only recognized event was receiving the Tdap vaccine one week before his illness.

## Conclusion

To the best of our knowledge, this is the first case of GBS to be reported in the English literature after administration of a Tdap vaccine.

## Competing interests

The author declares that they have no competing interests.

## Consent

Written informed consent was obtained from the patient for publication of this case report and any accompanying images. A copy of the written consent is available for review by the Editor-in-Chief of this journal.
